# High Prevalence and Lineage Diversity of Avian Malaria in Wild Populations of Great Tits (*Parus major*) and Mosquitoes (*Culex pipiens*)

**DOI:** 10.1371/journal.pone.0034964

**Published:** 2012-04-10

**Authors:** Olivier Glaizot, Luca Fumagalli, Katia Iritano, Fabrice Lalubin, Juan Van Rooyen, Philippe Christe

**Affiliations:** 1 Museum of Zoology, Lausanne, Switzerland; 2 Department of Ecology and Evolution, University of Lausanne, Lausanne, Switzerland; 3 Laboratory for Conservation Biology, Department of Ecology and Evolution, University of Lausanne, Lausanne, Switzerland; Institut Pasteur, France

## Abstract

Avian malaria studies have taken a prominent place in different aspects of evolutionary ecology. Despite a recent interest in the role of vectors within the complex interaction system of the malaria parasite, they have largely been ignored in most epidemiological studies. Epidemiology of the disease is however strongly related to the vector's ecology and behaviour, and there is a need for basic investigations to obtain a better picture of the natural associations between *Plasmodium* lineages, vector species and bird hosts.

The aim of the present study was to identify the mosquito species involved in the transmission of the haemosporidian parasites *Plasmodium* spp. in two wild populations of breeding great tits (*Parus major*) in western Switzerland. Additionally, we compared *Plasmodium* lineages, based on mitochondrial DNA cytochrome *b* sequences, between the vertebrate and dipteran hosts, and evaluated the prevalence of the parasite in the mosquito populations. *Plasmodium* spp. were detected in *Culex pipiens* only, with an overall 6.6% prevalence. Among the six cytochrome *b* lineages of *Plasmodium* identified in the mosquitoes, three were also present in great tits. The results provide evidence for the first time that *C. pipiens* can act as a natural vector of avian malaria in Europe and yield baseline data for future research on the epidemiology of avian malaria in European countries.

## Introduction

Transmission of avian malaria is highly dependent on the distribution, competence and vectorial capacity of vectors. Despite a recent increase in interest toward dipteran insects as major actors in avian malaria systems [1]–[4], there is a lack of general knowledge of the role of the vectors in this mosquito-borne disease. Most of the information on the vectorial competence of diverse mosquito species has been reviewed by Huff [5] but natural avian malaria vectors have been mostly ignored until very recently [6]–[8]. Although many species seem to be competent according to laboratory experiments [6], only a few recent studies have identified natural vectors responsible for avian malaria transmission in different parts of the world, with the notable exception of Europe [1], [2], [9]–[16].

Molecular studies on avian malaria have permitted the investigation of questions concerning phylogeny, phylogeography and life-history evolution of *Plasmodium* parasites [17]–[21]. However, the epidemiological success of *Plasmodium* strongly depends on its life cycle within the mosquito and in the vector's ability to find suitable hosts for taking a blood meal. Furthermore, asexual reproduction of *Plasmodium* occurs in vertebrate hosts whereas sexual reproduction takes place in the mosquito, making the vector the definitive host [22]. Mosquitoes are thus expected to play a major role in both avian malaria parasites' evolution and disease transmission [23], [24]. It is therefore of prime interest to deepen the understanding of the interactions between the different actors of this complex host-parasite system under wild conditions, the first step being the identification of the vector in such wild populations.

The aims of the present study were to identify the natural vector of avian malaria in two wild populations of great tits (*Parus major*) in Switzerland, where the prevalence of infection has been shown to be high [20], and to identify and compare malaria lineage diversity among the bird hosts and the mosquito vectors by means of mitochondrial cytochrome *b* gene sequencing.

## Materials and Methods

### Ethical notes

All necessary permits were obtained for the described field studies and the study was approved by the «Conservation de la Faune du Canton de Vaud». No other specific permissions were required for this study and study sites were non-protected, public properties. This study did not involved endangered or protected species. All birds were treated in accordance with the Cantonal Veterinary Authorities of the Canton de Vaud, Switzerland, authorization 1730. Captures of birds were made under license number F044-0799 of the Swiss Federal Office for the Environment.

### Study sites

The study was carried out in 2006 and 2007 in two populations of great tits (*Parus major*) breeding in nestboxes. The first population was located in the forest of Dorigny, on the campus of the University of Lausanne (46°31′N; 6°34′E; alt. 400 m). This forest is mainly composed of beech (*Fagus sylvatica*) and a mixture of large dominant deciduous species like oak (*Quercus*) and hornbeam (*Carpinus*). The second study area was located in the forest of Monods (46°34′N; 6°24′E; alt. 680 m). This mixed forest is composed predominantly of beech and spruce (*Picea abies*) and surrounded by an adler (*Alnus glutinosa*) forest flooded with stagnant water.

### Sampling of mosquitoes

Mosquitoes were sampled from July to September, using both BG-Sentinel traps (BioGents GmbH, Germany) and gravid mosquito traps (Bioquip, California). The first device, using CO_2_ (dry ice) as a lure, was used to collect host-seeking mosquitoes whereas gravid mosquito traps were intended to capture fed females just before or after oviposition. As egg laying usually takes place five (up to ten) days after blood feeding [25], complete digestion should have occurred at the time of oviposition ensuring absence of remnant bird blood in the digestive tract. As a precaution, however, female mosquitoes were kept alive for 48 hours after trapping before being killed. Mosquito species were identified by morphology in the laboratory.

### Sampling of birds

Adult great tits were trapped in their nestboxes during the breeding season by using door traps mounted inside the nestboxes when their nestlings were fourteen days old. A blood sample (20 µl) was then taken by brachial venapuncture and stored at −20°C until molecular assessment of malarial lineages.

### Molecular analyses

DNA was extracted from thoraxes of unfed mosquitoes and from adult bird blood samples using the DNeasy tissue extraction kit (QIAGEN) according to the manufacturer's protocol, and was resuspended in 200 µl TE buffer.

Parasite lineages were detected by using a nested PCR method developed by Waldenström et al. [26] from the original protocol [17], amplifying a portion of the mitochondrial DNA (mtDNA) cytochrome *b* gene. Full details of PCR and sequencing conditions are described in Christe et al. [20]. Sequences were edited and aligned using the program SEQUENCHER 3.0 (Gene Codes, Ann Arbor, Michigan) with additional manual editing. Mitochondrial DNA lineages were identified by comparison with published sequences available on GenBank, and named according to the MalAvi database [27]. Sequences overlapping with less than the 478-bp reference sequence of the MalAvi database were assigned to a precise haplotype name only if they were identical to a reference sequence present in the database. For linkage between mtDNA lineages and morphospecies, we referred to studies combining molecular and morphological approaches [28]–[30] as well as publications matching cytochrome *b* haplotypes to a sequence in GenBank associated with a particular morphotype. Sequences were deposited in GenBank (Accession Nos. JQ778276–JQ778282).

## Results

Six genera of mosquitoes representing a total of 15 species were captured from the study populations. Among them, nine species (four genera) are mainly or potentially ornithophilous and were analyzed for the presence of *Plasmodium* and *Haemoproteus* parasites (Table 1). Only *Culex pipiens* individuals were found to be positive for avian malaria with an overall prevalence of 6.6% (n = 394).

**Table 1:** Analyses of ornithophilous mosquito species per study location.

	Dorigny		Monods		Overall	
Species	N (+)	%	N (+)	%	N (+)	%
*Anopheles maculipennis*	-	-	2 (0)	0	2(0)	0
*Coquillettidia richiardii*	1 (0)	0	-	-	1(0)	-
*Culex hortensis*	1 (0)	0	-	-	1 (0)	-
*Culex pipiens*	330 (26)	7.9	64 (0)	0	394 (26)	6.6
*Culex torrentium*	1 (0)	0	-	-	1 (0)	-
*Culiseta alaskaensis*	1 (0)	0	24 (0)	0	25 (0)	0
*Culiseta annulata*	5 (0)	0	3 (0)	0	8 (0)	0
*Culiseta fumipennis*	-	-	2 (0)	0	2 (0)	0
*Culiseta morsitans*	-	-	31 (0)	0	31 (0)	0

The number of screened specimens (N), the number of positive samples (+) as well as the prevalence (%) is given.

All infected *C. pipiens* originated from the Dorigny forest (26 out of 330 individuals collected, 7.9%), whereas none of the 64 collected in the Monods forest were found to be infected. Proportions of infected birds at both locations were comparable, with 91% of infected birds in the Monods forest and 98% in Dorigny.

Six different *Plasmodium* lineages were identified among the 26 infected *Culex pipiens*, and four among the 54 great tits analysed (Table 2). One lineage of *Haemoproteus*, known to be transmitted by non-mosquito vectors, was found in a great tit individual.

**Table 2:** *Plasmodium* and *Haemoproteus* lineages detected by sequencing a portion of the mtDNA cyt *b* gene (433–478 bp) in 54 *Parus major* and 26 *Culex pipiens* individuals.

Lineages	Reference	GenBank accession no.	Morphospecies	No. of infected birds	No. of infected mosquitoes
SGS1	[17]	AF495571	*Plasmodium relictum*	33	6
GRW11	[35]	AY831748	*P. relictum*	0	2
P5	[36]	DQ838991	*P. relictum*	0	2
TURDUS1	[37]	AF495576	*P. circumflexum*	7	5
SW2	[37]	AF495572	*P. polare*	13	5
SYAT05	[19]	DQ847271	*P. vaughani*	0	6
PARUS1	[17]	AF254977	*Haemoproteus majoris*	1	0
**TOTAL**				**54**	**26**

## Discussion

The relatively high prevalence of *Plasmodium* spp. found in females *Culex pipiens* strongly suggests that this species acts as a vector of avian malaria in one of our study populations. *C. pipiens* was infected with six *Plasmodium* lineages, among which three were also identified in great tits. *Haemoproteus* spp., transmitted by biting midges of the genus *Culicoides* [31], was present in one great tit individual.

Many species of *Culex* have been described as vectors of avian malaria parasites and their role as competent vectors in experimental infections has been proven [32]. *C. pipiens*, or the two subspecies *C. p. pipiens* and *C. p. pallens*, have been recently reported to be the natural vector of avian malaria in Japan [1], [10]–[14] and North America [2]. Other species of the genus *Culex* have been described as vectors of avian *Plasmodium*: *C. restuans* in North America [2], [16], *C. (Melanoconion) ocossa* in Panama [8], *C. saltanensis* in Brazil [33], *C. sasai* in Japan [14], *C. quinquefasciatus* in Japan [12] Hawai'i [34] and Mexico [15], *C. neavei*, *C. perfidiosus*, *C. poicilipes* and *C. guiarti* in Cameroon [35] and finally *C. sitiens* and *C. annulirostris* in the southwest Pacific islands [9]. The results of the present study suggest therefore for the first time the role of *Culex pipiens* in the transmission of avian malaria in the European continent.

None of the mosquitoes captured in Monods were positive for malaria, although prevalence in great tits was 91%. This could be due to our low sample size of parous females and/or to other vector species involved in the transmission of *Plasmodium* present in this particular site. *Culiseta morsitans* has been shown to be the vector of *Plasmodium circumflexum* in waterfowl in New Brunswick [36], whereas Kimura et al. [2] did not find any *Plasmodium* in 176 *Culiseta melanura* in Ithaca (USA). Furthermore, *C. morsitans* was also described as competent vector of *P. polare* [6], which presents an overall prevalence of 24% and 19.2% among great tits and infected *C. pipiens*, respectively. The low number of *Culiseta morsitans* (n = 31) sampled in the present study did not permit the exclusion of this species as potential vector and ongoing studies should elucidate this issue.

Mosquito samplings may not reflect the relative abundances of species in the study sites. First, the type of traps or attractant used may be specific to one or several species [37]. Secondly, capture success of a given species may depend on the trap location itself. A recent study found a higher capture rate for *C. pipiens* at ground compared to canopy level, whereas biting midges (*Culicoides* spp.) (Diptera: Ceratopogonidae), known to be involved in the transmission of *Haemoproteus*, showed the opposite pattern [38]. Similarly, the opportunistic *Anopheles plumbeus* showed a spatial preference for canopy level [38]. In the present study, gravid mosquito traps were placed at ground level whereas sentinel traps caught mosquitoes approximately 3 meters from the ground. An ongoing study on overall mosquito diversities (Glaizot, unpublished) revealed that *C. pipiens* was the most caught mosquito species in the Dorigny population (723/953 = 75.9%) whereas it represents 12.1% of the captures in Monods (150/1237). In this latter population, the other main ornithophilic species (*Culiseta alaskaensis* and *C. morsitans*) were captured at a rate of 7.1% and 5.2%, respectively. A bias in the trapping method cannot be excluded and further studies should also concentrated on mosquito trapping at the canopy level. Apart strict ornithophilic species listed in Table 1, some mammophilic and opportunistic species are abundant in the study populations. For example, *Anopheles plumbeus* is widespread in the Dorigny population (136/953 = 14.3%) whereas the mammophilic *Ochlerotatus rusticus* (579/1237 = 46.8%) and *Ochlerotatus annulipes/riparius/cantans* (283/1237 = 22.9%) are the most abundant species in Monods.

In the present study, parasite DNA was extracted from dissected mosquito thoraxes. One potential problem to this approach is that sporozoites, the ultimate parasite life stage before transmission to birds, can occur in the haemocoel during their travel from the midgut to the salivary glands but never reach and fully develop in the salivary glands. Consequently, the amplified parasite DNA from mosquito thoraxes may come from non-infectious parasite stages, and therefore non competent mosquitoes [39]. Experimental infections and/or microscopic detection of both oocysts in the midgut and sporozoite in the salivary glands would fully confirm the role of *C. pipiens* as a natural vector in the study populations. However, the absence of *Plasmodium* DNA in the other mosquito species captured and the presence of three lineages found in both birds and mosquitoes strongly suggests that *C. pipiens* is an active actor for the transmission of *Plasmodium* spp to *Parus major*. Moreover, an ongoing study (Lalubin *et al.*, unpublished), which combines molecular detection methods with morphological examination of mosquito midguts and salivary glands, confirms the role of *C. pipiens* in the transmission cycle of avian malaria.

*P. relictum* (lineages SGS1, GRW11 and P5) was the most abundant haemosporidian parasite found in the great tit populations and one of the main morphospecies responsible for infection in *C. pipiens* at Dorigny, a result similar to those reported for Japan [1]. In laboratory conditions, *P. relictum* develops in more than 20 mosquito species including *C. pipiens* [32] belonging to six genera and has a large bird host range including more than 300 species from different orders and a worldwide distribution [6]. *P. polare* (SW2) was well represented both in the bird and mosquito samples. According to Valkiunas [6], this parasite has been recorded among 22 bird species in all zoogeographical regions except Antartica and Australia. However, to our knowledge, the present study is the first to report its presence in *C. pipiens*. Another common lineage, TURDUS 1, assigned to the morphospecies *P. circumflexum*, has also been found both in great tits and, for the first time, in *C. pipiens*. This morphospecies has been recorded in over 100 bird species across all regions except the Antartic [6]. Finally, lineage SYAT05, assigned to *Plasmodium vaughani*, has been found in more than 200 Passeriformes as well as other bird orders [6]. *C. pipiens and C. restuans* have been reported as vectors for this lineage [2].

Knowledge on temporal and spatial patterns of parasite transmission is essential to understand the selective pressure imposed by parasites on their hosts. For the first time described as a natural vector of avian malaria in Europe, *Culex pipiens* seems to play a major role in the transmission of a high range of *Plasmodium* morphospecies. The higher diversity of lineages of *Plasmodium* found in *C. pipiens* compared to those found in great tits also suggests that *C. pipiens* is a natural avian malarial vector for several other bird species. Furthermore, the presence of some of these *Plasmodium* lineages in bird species sampled in Africa [40] suggests that in these migrating species, infection may also occur in Europe.

Most of the malarial studies to date have used non-natural systems (i.e. hosts and parasites that would not encounter one another in nature). Using vertebrate/mosquito/haemosporidian model systems from natural populations of wild species [41]–[43] may provide powerful insights into the complex interactions and constraints affecting the relationship between hosts and parasites.
